# Associations Between Sleep Patterns, Circadian Preference, and Anxiety and Depression: A Two-Year Prospective Study Among Norwegian Adolescents

**DOI:** 10.3390/clockssleep7020026

**Published:** 2025-05-27

**Authors:** Linn Nyjordet Evanger, Ingvild West Saxvig, Ståle Pallesen, Michael Gradisar, Stein Atle Lie, Bjørn Bjorvatn

**Affiliations:** 1 Department of Global Public Health and Primary Care, University of Bergen, 5020 Bergen, Norway; 2 Norwegian Competence Center for Sleep Disorders, Haukeland University Hospital, 5021 Bergen, Norway; ingvild.west.saxvig@helse-bergen.no (I.W.S.); staale.pallesen@uib.no (S.P.); 3 Department of Psychosocial Science, University of Bergen, 5020 Bergen, Norway; 4 Sleep Cycle AB, Sleep Science Team, 412 50 Gothenburg, Sweden; grad0011.mg@gmail.com; 5 Centre for Translational Oral Research, Department of Clinical Dentistry, University of Bergen, 5020 Bergen, Norway; stein.lie@uib.no

**Keywords:** adolescents, sleep duration, insomnia, depression, anxiety

## Abstract

This study explored whether sleep duration, insomnia, social jetlag, and circadian preference predicted adolescents’ risk of anxiety and depression two years later. High school students initially aged 16–17 years were, in 2019 and 2021, invited to a web-based survey assessing sleep patterns, insomnia, circadian preference, anxiety, and depression. Sleep duration, insomnia, circadian preference, depression, and anxiety were assessed using the Munich ChronoType Questionnaire, the Bergen Insomnia Scale, the reduced Morningness–Eveningness Questionnaire, the Patient Health Questionnaire-9, and the Generalized Anxiety-Disorder 7, respectively. Analyses were conducted using logistic regression analyses. The analytic longitudinal sample comprised 1456 students (initial mean age 16.4 years; 61.4% girls). Short school night sleep duration, chronic insomnia, and more severe insomnia symptoms at baseline predicted greater risk of anxiety and depression at follow-up when controlled for anxiety and depression at baseline. Neither free night sleep duration nor social jetlag at baseline were related to the risk of anxiety and depression at follow-up. When circadian preference was investigated continuously, greater morningness at baseline predicted lower risk of anxiety and depression at follow-up. When circadian preference was investigated categorically, evening preference type was associated with higher risk of depression at follow-up than intermediate preference type, while the prospective risk of anxiety and depression otherwise did not differ in relation to circadian preference. The results attest to prospective associations between adolescent sleep problems at baseline and later risk of anxiety and depression.

## 1. Introduction

Adolescence is an important period with respect to social, neurocognitive, and emotional development [1,2,3], and adequate sleep is pivotal for adolescents’ health and daytime functioning [4,5]. Despite this, poor and insufficient sleep represents wide-spread phenomena in adolescent populations across the globe [6,7]. It is well established that sleep disturbances may increase the risk of developing anxiety and depression (collectively often referred to as “internalizing disorders”), which often mark their debut during adolescence [3,8,9]. As both sleep problems and internalizing disorders among adolescents have been linked to adverse developmental outcomes, including dropout from school [10,11], an adequate understanding of how disturbed sleep is related to internalizing disorders is warranted to optimize adolescents’ developmental trajectories.

Building on available evidence, the prevailing theoretical framework for understanding short sleep duration among adolescents proposes that both homeostatic and circadian changes during puberty interact with various psychosocial changes and behavioral factors (e.g., social media use [12]), resulting in a delay in the timing of sleep [13,14]. Consistent with this, adolescents exhibit gradually later circadian preference (i.e., late preferred timing for sleep and activity) [15]. Since school tends to start relatively early in the morning, these changes result in reduced opportunities for sleep [14,16]. In line with this, several recent large-scale studies have shown that the majority of adolescents tend to sleep far below the 8–10 h recommended for adolescents aged 14–17 years [17,18,19]. Also, short sleep duration is primarily a school night phenomenon, with several studies showing that the duration is often within the recommended range on free nights [5,6,7,19]. The mismatch between the biological clock and social demands, known as “social jetlag”, is typically defined as the discrepancy in sleep timing on school versus free nights [20]. Social jetlag is normally larger among adolescents with later circadian preference and appears to be perpetuated by weekend “catch-up sleep” following sleep debt on weekdays [21,22,23]. While social jetlag has been positively associated with anxiety and depression in some studies [21,24], it is currently unclear whether social jetlag precedes these mental disorders [25].

Sleep disorders represent another potential cause of insufficient sleep during adolescence, with the most prevalent being insomnia [26,27]. Insomnia encompasses experienced difficulties in initiating and/or maintaining sleep to a degree that causes sleep-related worry and/or functional difficulties in private, occupational, or educational domains [26,28]. Distinguishing insomnia from other causes of poor sleep is challenging due to symptomatic overlap [29]; however, estimates suggest that 4–39% of adolescents qualify for the disorder [27,30]. Girls are more often affected than boys [27,31]. Insomnia disorder is often chronic [31,32], assumably due to a combination of predisposing factors and maladaptive ways of coping with the symptoms [33,34].

It is well established that psychiatric symptoms are more prevalent among adolescents with poor or insufficient sleep than among good-sleeping adolescents [33,35]. For example, a large-scale study from the US found that approximately half of adolescents with insomnia qualified for at least one co-existing psychiatric condition [31]. Sleep disturbances are further associated with suicidal attempts and ideations in adolescents [36]. Sleep disturbances in the presence of depression, anxiety, or other psychiatric disorders were historically attributed to the psychiatric disorder and, hence, regarded as symptoms/consequences of the other condition [37,38]. Although sleep problems may manifest as a symptom of internalizing disorders, it is now evident that adolescent sleep problems are often bidirectionally related to depression and anxiety, although not all studies have reported such associations [39,40,41]. For example, one study found that poor sleep predicted a later increase in anxiety symptoms in early and mid-adolescence, while the reverse association was not supported [42].

Despite evidence that poor sleep often precedes internalizing disorders, few have looked at whether social jetlag and later circadian preference precede these disorders in adolescents specifically [25,39]. Also, although adolescents with later circadian preference tend to sleep less on school nights than those with an earlier preference (by one hour, according to one of our previous publications [23]), few previous studies have investigated the longitudinal associations between circadian preference and internalizing disorders, according to a recent review [39]. While two studies included in the review reported that morning preference at baseline was associated with lower symptoms of depression and anxiety over time [43,44], a third study found no such association [45], leaving the overall evidence of longitudinal associations between circadian preference and internalizing disorders unclear [39]. Also, few studies have previously investigated whether social jetlag is related to an increased risk of developing anxiety and depression over time [25,46].

Based on the Western Norway Longitudinal Sleep Study [19], the current study aimed to explore the prospective relationships between sleep duration, insomnia, social jetlag and circadian preference on adolescents’ risk of having anxiety or depression two years later. As short sleep duration is primarily a school night phenomenon, it was expected that shorter school night sleep duration at baseline would predict an increased risk of having anxiety and depression at follow-up (hypothesis 1). However, given that adolescents’ sleep duration is often within the recommended range on free nights, the same associations were not expected on free nights (hypothesis 2). Third, we expected that chronic insomnia and more severe insomnia symptoms at baseline would be associated with an increased risk of anxiety and depression at follow-up (hypothesis 3). Furthermore, we predicted that more social jetlag and later circadian preference at baseline would be associated with a higher risk of developing anxiety and depression from baseline to follow-up (hypothesis 4).

We also investigated the inverse associations between internalizing disorders at baseline and sleep duration on school nights, chronic insomnia, social jetlag and circadian preference at follow-up. Given previous studies that sleep and internalizing disorders are often bidirectionally related in adolescent cohorts [39,40,47], we predicted that students screening positive for anxiety and depression would have a lower risk of sleep duration ≥ 8 h as well as greater risk of having chronic insomnia and social jetlag ≥ 2 h at follow-up when controlling for the corresponding sleep parameters at baseline (hypothesis 5).

## 2. Results

Demographics are presented in Table 1. Comparisons regarding sleep and mental health characteristics between baseline and follow-up in the final longitudinal sample are presented in Table 2. The average school night sleep duration at baseline was 6 h and 49 min, with a slight increase of 6 min at follow-up (*p* < 0.001). At baseline, 33.0% of the students screened positive for chronic insomnia, with a slight but insignificant increase to 35.4% at follow-up (*p* = 0.132; Table 2). Also, 27.6% screened positive for anxiety at baseline, and the rate increased significantly to 33.5% at follow-up (*p* < 0.001). The proportion of depression at baseline was 27.8%, with a significant increase to 35.9% at follow-up (*p* < 0.001). The majority (53.0%) of the students were categorized as intermediate circadian preference types at baseline, followed by 37.3% categorized as evening types, whereas only 9.7% were categorized as morning types. Investigated continuously, circadian preference was slightly but significantly advanced from baseline to follow-up (*p* < 0.001). Social jetlag averaged 2 h and 36 min at baseline, with a small but significant decrease by 8 min at follow-up (*p* < 0.001). In total, 73.9% of the sample reported social jetlag ≥ 2 h, and the proportion decreased to 67.6% at follow-up (*p* < 0.001; Table 2). The rate of participants using prescribed sleep medications at least weekly increased slightly but significantly from 2.5% at baseline to 3.8% at follow-up (*p* = 0.030). The average units of caffeinated beverages per day increased from 2.0 at baseline to 2.3 (both *SD* = 1.5) at follow-up (*p* < 0.001; *t* = −5.26).

Regarding the ongoing COVID-19 pandemic, the majority (71.4%) reported not having remote teaching at the time the follow-up study was conducted. Additionally, 53.1% reported never having had remote teaching during the past two weeks, while 22.4% of the students reported having had remote teaching from home for three days or less. A minority (15.4%) had had remote teaching for all school days during the past two weeks.

For baseline associations between sleep patterns, circadian preference, social jetlag and anxiety and depression, please see Table S1 in the Supplementary Section.

### 2.1. Sleep Patterns, Circadian Preference, and Social Jetlag as Longitudinal Predictors of Anxiety and Depression

Longitudinal associations are presented in Table 3. Adjusted for anxiety and depression at baseline, each additional baseline hour of sleep on school nights predicted 16% lower odds of anxiety and 18% lower odds of depression at follow-up. Furthermore, both measures of insomnia at baseline were associated with higher odds of both anxiety and depression at follow-up. Specifically, students with chronic insomnia at baseline had 1.86 higher odds of anxiety and 1.82 higher odds of depression at follow-up. For each increased score on the BIS, the odds of anxiety increased by 6%, while the odds of depression increased by 7%. By contrast, no associations were found between hours of reported sleep on free nights at baseline and the odds of either anxiety or depression at follow-up (Table 3).

Each increased unit on the rMEQ, indicating greater tendency toward morningness, predicted 5% lower odds of depression. rMEQ was, however, unrelated to the longitudinal trajectory of anxiety (Table 3). Investigated categorically, no differences in the risk of either anxiety or depression were observed in relation to circadian preference when adjusted for anxiety/depression at baseline. Furthermore, no associations were found between social jetlag at baseline and the odds of either anxiety or depression at follow-up.

#### Adjusted Analyses

Longitudinal associations adjusted for sex and maternal education are presented in Table 3. More hours of sleep on school nights still predicted lower odds of anxiety and depression at follow-up, whereas chronic insomnia and more severe insomnia symptoms at baseline still predicted increases. A higher rMEQ score at baseline (indicating more morningness) still predicted decreased odds of depression at follow-up and additionally predicted decreased odds of anxiety at follow-up. Categorically, students with evening preference at baseline had greater odds of depression at follow-up than those with an intermediate preference; otherwise, the odds of anxiety and depression did not differ in relation to circadian preference. Social jetlag at baseline remained unrelated to the odds of both anxiety and depression at follow-up.

All associations mostly remained the same when investigated continuously. However, more minutes of social jetlag predicted increased symptoms of depression over time when controlled for sex and maternal education.

### 2.2. Anxiety/Depression as Predictors of Sleep Duration, Insomnia, and Circadian Preference

Reverse analyses investigating anxiety and depression at baseline as longitudinal predictors of sleep duration ≥ 8 h on school and free nights, chronic insomnia, circadian preference (morning and intermediate against evening), and social jetlag ≥ 2 h are presented in Table 4. Anxiety and depression at baseline were both associated with higher odds of chronic insomnia and, surprisingly, with lower odds of social jetlag ≥ 2 h at follow-up when adjusted for chronic insomnia and social jetlag ≥ 2 h at baseline. Students with baseline depression also had greater odds of evening preference at follow-up when controlling for circadian preference at baseline.

## 3. Discussion

The present study aimed to investigate whether sleep duration, insomnia, social jetlag, and circadian preference predicted older adolescents’ risk of anxiety and depression two years later. This study showed that longer school night sleep duration at baseline reduced adolescents’ risk of anxiety and depression two years later. Reversely, chronic insomnia and more severe insomnia symptoms at baseline predicted an increased risk of anxiety and depression at follow-up. Neither free night sleep duration (e.g., weekend sleep) nor social jetlag was related to the risk of anxiety and depression over time. Higher tendency toward morningness predicted a decreased risk of depression over time and decreased risk of anxiety over time when maternal education and sex were taken into consideration. When analyzing data categorically, students with evening preference had a greater risk of depression at follow-up than intermediate types but only when we controlled for sex and maternal education. The prospective risk of anxiety and depression did not otherwise differ across the circadian preference types.

The finding that longer school night sleep duration at baseline predicted a reduced risk of anxiety and depression at follow-up supported hypothesis 1, implying that less sleep on school nights predicted poorer mental health over time. However, in support of hypothesis 2, sleep duration on free nights was unrelated to changes in the risk of anxiety and depression over time. This lack of associations was expected given that sleep deprivation among adolescents is primarily a school night phenomenon [5,19]. The symptoms resulting from insufficient sleep can overlap with emotional disorders, such as depression (i.e., low motivation and energy, fatigue, tiredness, impaired concentration) [48], which may explain why poor sleep may trigger the onset of later emotional disorders.

The findings that both chronic insomnia and more severe insomnia symptoms at baseline predicted a greater risk of anxiety and depression at follow-up supported hypothesis 3. While insomnia has historically been conceptualized as a symptom of anxiety and depression [37,38], the current results support the past decades of research, suggesting that insomnia may represent a risk factor for developing anxiety and depression over time [49,50]. It should, however, be noted that the BIS is highly sensitive in terms of detecting insomnia but, to a lesser extent, able to differentiate insomnia from other causes of poor sleep, which may have affected the insomnia assessments in the current study due to the tendency of adolescents’ circadian patterns to be delayed. This was demonstrated in a recent publication from the initial 2019 wave of the WALOSS study, where 88.4% of the students who met the criteria for insomnia based on the BIS presented at least one symptom of circadian delay, while 15.5% of the students presented four of four symptoms of circadian delay [51]. On the other hand, the insomnia rate of 33% at baseline in the current study indicates that the proportion of adolescents struggling with sleep to a problematic degree is nevertheless high. As insomnia is common, often chronic, and often underdiagnosed and undertreated [28,29,32,52], the results, therefore, indicate that addressing adolescent insomnia is important to prevent mental health complaints in this age group.

Contrary to hypothesis 4, social jetlag was unrelated to the risk of anxiety and depression at follow-up, indicating that irregular sleep timing between school and free nights did not increase adolescents’ risk for developing anxiety and depression in the present study. Possible explanations for these non-significant associations are unclear, as weekend catch-up sleep may lead to further delays in adolescents’ sleep timing and generally reflects greater sleep debt on weekdays. Given that few studies have explored the prospective associations between social jetlag and internalizing disorders previously [25,46], we will argue that more research in this field is necessary.

The hypothesized links between later circadian preference and the students´ prospective risk of anxiety and depression were only partly supported. While greater tendency toward morningness predicted a decreased risk of anxiety and depression over time when investigated on a continuous basis, the longitudinal risk of anxiety and depression mostly did not otherwise differ in relation to circadian preference. The lack of clearer longitudinal associations between circadian preference and anxiety and depression was unexpected, for several reasons. For example, previous studies suggest that adolescents with later circadian preference sleep for substantially less time on school nights than adolescents with early circadian preference [23,53]. As shorter sleep duration on school nights was associated with greater risk of anxiety and depression in the present study, it was, therefore, surprising that circadian preference was unrelated to changes in the risk of anxiety and depression over time. One likely explanation behind the non-significant findings could be that adolescents with morning-type preference also tend to report insufficient sleep duration on school nights, according to a previous publication on the same study [23]. Furthermore, it should be noted that circadian preference reflects an individual’s preferred timing for sleep rather than the individual’s current/actual sleep–wake patterns (known as chronotype [23]), meaning that the students’ circadian preference did not necessarily reflect their current sleep patterns in our sample. Also, it should be noted that the slight change toward more morningness over time observed in the present study contrasts with several previous studies, suggesting a peak of eveningness at the approximate age of 20, although this may vary across studies [15,54]. For example, a study by Randler (2011) found that, although circadian preference remained stable from the ages of 15 to 20 after changing toward eveningness between the age of 12 and 15, a change toward greater morningness was observed first at the age of 21 [54]. Another possible explanation behind the current results is that the students were in their final high school year, meaning that their academic pressure might have been higher than before. Also, being in their last high school year might have encouraged them to put more emphasis on obtaining good grades. Both these factors would likely motivate them to advance their circadian rhythm. Nevertheless, it remains uncertain why late circadian preference was partly related to an increased risk of depression and anxiety, while social jetlag was unrelated to the corresponding risks. As relatively few studies have previously investigated the prospective associations between circadian preference and the risk of internalizing disorders, more research is needed.

### 3.1. Implications

As a substantial number of adolescents are sleep deprived on school nights [6], the findings suggest that interventions seeking to improve adolescents’ sleep duration may help reduce their prospective risk of developing internalizing disorders. In line with this, recent meta-analyses have shown that later school start times, allowing adolescents to follow their natural sleep timing, are related to longer sleep duration and less negative mood [16,55]. The results also highlight the importance of adolescents and their parents to prioritize sleep and practice good sleep hygiene, which may be particularly warranted due to the adolescents’ natural preferences for late sleep timing [14]. Finally, the prospective associations between insomnia and increased risk of anxiety and depression indicate that addressing adolescent insomnia is important, especially as insomnia is a relatively persistent and untreated condition [27,31,32].

This study also supports the notion of bidirectional associations between insomnia and internalizing disorders, respectively, which are in line with many previous studies [39]. This suggests the need for a holistic approach to ensure optimal health and well-being among older adolescents.

### 3.2. Strengths and Limitations

This study has several strengths. A major strength is the longitudinal design, allowing exploration of a temporal relationship between sleep and mental health. Also, the large sample size of 1456 students enhances the study’s generalizability. Methodologically, this study relied on validated self-report questionnaires in assessing sleep-related characteristics, anxiety and depression, which increases the study’s internal validity.

Several limitations should, however, also be noted. First, the assessments relied on self-report screening instruments in assessing insomnia, depression and anxiety rather than clinical interviews, as it would not be practically feasible to conduct clinical interviews on the high number of participants originally invited to partake in this study. Thus, the cases falling into the categories of clinical depression, anxiety, or chronic insomnia should be considered as screening positive rather than necessarily reflecting true clinical cases. Second, this study did not include any objective sleep assessments. Thus, we cannot rule out the possibility that the results would differ if such measures were used instead. In support of this, a meta-analysis by Bacaro and colleagues found that the associations between sleep duration and internalizing symptoms were stronger in studies relying on subjective rather than objective sleep measures [39]. Also, while assessing sleep duration based on the Munich ChronoType Questionnaire with an additional item assessing nocturnal awakenings was a strength of the present study, the fact that some response options were presented as 15 min intervals may have reduced the accuracy of the sleep duration calculations. For the abovementioned reasons, we will, for similar studies in the future, recommend the use of objective sleep assessments (i.e., actigraphy) if feasible. Likewise, the use of digital sleep diaries in future studies will likely reduce the potential risk of memory biases in reported sleep, which may have affected the current sleep estimates. Furthermore, as the invited schools were asked to prioritize one school hour to complete the survey and sleep, anxiety and depressive disorders in previous studies have been related to school absenteeism [56,57], the sample may have been underrepresented by students with these diagnoses. Also, the Cronbach’s alphas on the rMEQ of 0.56 and 0.60 at baseline and follow-up, respectively, were low. Cronbach’s alpha is highly sensitive to the number of items in a scale [58], which may partly explain the low values. While the low Cronbach’s alpha largely corresponds to a Swedish validation study of the rMEQ among youths aged 16–26 years [59], the low value nevertheless warrants caution when interpreting the results from this instrument. In future studies, we may, therefore, recommend use of the full version of the MEQ instead to see if this changes the results. Additionally, it should be noted that the data were collected during the COVID-19 pandemic. Although the students had normal school schedules in the baseline survey in 2019, and the spring semester of 2021 to a lesser extent was characterized by restrictions in Norway, the pandemic may nevertheless have affected the results. Thus, we recommend replication of the present study in a non-pandemic context. Finally, it should be noted that we did not have information about potential comorbid conditions other than the mental health complaints investigated in the present study. Similarly, we did not have information about whether the students received medications or psychotherapy for their mental health complaints, or whether they were under any form of sleep-promoting intervention (e.g., cognitive behavioral therapy for insomnia), under the study period.

## 4. Methods and Materials

The current study is based on data from the Western Norway Adolescent Longitudinal Sleep Study (WALOSS) [19], which was conducted annually in the period 2019–2021. All high school students in Hordaland and Rogaland counties were initially invited to respond to a web-based survey asking about the adolescents’ sleep habits, anxiety and depression symptoms, and demographics between April and May 2019. Students in Rogaland were invited through SMS or e-mail, whereas students in Hordaland were invited through their school’s electronic platform. Students who responded to the 2019 survey, and who were confirmed to be high school students by their respective school administrations, were re-invited to follow-up surveys during the spring of 2020 and 2021, respectively. Due to the COVID-19 outbreak in the late winter/spring of 2020, Norwegian high schools were closed and practiced remote teaching in the spring semester of 2020. In the school year 2020–2021, the students faced periods with various and shifting restrictions, both at local and national level, and schools were instructed to practice remote teaching when the infection pressure was high. To minimize potential impacts of the COVID-19 pandemic, the present study only used data collected in 2019 and 2021, respectively.

### 4.1. Instruments

#### 4.1.1. Sleep Duration and Social Jetlag

Sleep duration on school and free nights during the past four weeks was assessed using a Norwegian version of the Munich ChronoType Questionnaire for children and adolescents (MCTQ) [60]. The MCTQ assesses self-reported sleep on week and free nights separately based on the following sleep parameters: bedtime (BT), time of preparing to sleep (SPrep), sleep onset latency (SLat), and sleep end (SE). The survey also comprised an additional item targeting duration of nocturnal awakenings (WASO) on school and free nights, asking “For how long are you awake during the night on school-/free days?”

All response options were available through drop-down menus. All clock time items (BT, SPrep and sleep end) were presented as 15 min interval scales. Items assessing duration (SLat and WASO) were presented as 5 min interval scales within the range “0 min” to “5 h or more”. Option ranges for BT and SPrep were presented between “20:00 hours or earlier” and “08:00 hours or later”, whereas SE was presented in the range “05:00 hours or earlier” to “17:00 hours or later”. Hours of sleep duration on school and free nights, respectively, were calculated as time in bed extracted for wakefulness in bed. Social jetlag was calculated as the midpoint of sleep on free nights minus the midpoint for sleep on school nights.

#### 4.1.2. Circadian Preference

Circadian preference was assessed using the reduced version of the Morningness–Eveningness Questionnaire (rMEQ) [61,62], translated into Norwegian. The rMEQ includes five items related to preferences for sleep and daily activities. The possible total score ranges from 4 to 25, with higher score indicating greater morningness tendency. In the present study, we used the following cut-off criteria: morning preference (sum score > 17); intermediate preference (sum score 12 to 17); or evening preference (sum score < 12) [61]. Cronbach’s alpha for the total continuous scale was 0.56 at baseline and 0.60 at follow-up.

#### 4.1.3. Insomnia

Insomnia was assessed using a Norwegian version of the Bergen Insomnia Scale (BIS), a 6-item scale validated for assessing insomnia symptoms [63]. The BIS assesses how many days weekly, on average, the three core symptoms of insomnia have been present during the past three months (sleep onset > 30 min; nocturnal awakenings > 30 min; morning awakening > 30 min before desired time), as well as an additional item targeting insufficient sleep quality. The scale also assesses how many days per week the respondent has experienced excessive sleep-related worry or sleepiness to a degree that has interfered with the respondents’ daily functioning within the occupational, educational, and/or social domains. Scoring options range from 0 to 7 days a week.

To meet the criteria for chronic insomnia, BIS requires that one or more of the three core symptoms have been experienced at least three times weekly over the past three months. Also, the respondent needs to report either sleep-related dissatisfaction/worry and/or daytime impairments at least three times weekly over the same period. It is also possible to use the scale continuously by summarizing the score of each item, yielding a composite score of 0–42, with higher score indicating more severe insomnia symptoms. The scale is widely used in research settings and has good psychometric properties, including in younger cohorts [63,64]. In the present study, Cronbach’s alpha was 0.77 at baseline and 0.79 at follow-up for the continuous scale.

#### 4.1.4. Anxiety

Anxiety was assessed using a Norwegian translation of the Generalized Anxiety Questionnaire-7 (GAD-7). The GAD-7 assesses to what extent seven common symptoms of anxiety have been present during the past two weeks. Scoring options are “not at all” (scored 0), “some days” (1), “more than half the days” (2), or “almost every day” (3), resulting in a possible total score of 0–21. Higher score indicates higher anxiety symptom severity. The scale can also be used diagnostically, with a cut-off of 8 or higher typically recommended to define clinical anxiety [65]. The Norwegian translation of the scale has demonstrated acceptable psychometric properties in the Norwegian population [66]. Cronbach’s alpha was 0.89 at baseline and 0.91 at follow-up for the continuous scale.

#### 4.1.5. Depression

Depression was assessed using a Norwegian translation of the Patient Health Questionnaire (PHQ-9). The PHQ-9 has been validated to detect clinical depression and depression symptom severity [67,68]. The official Norwegian translations of this questionnaire have previously been used and evaluated in different Norwegian samples, showing acceptable psychometric properties [66,69,70]. The PHQ-9 asks to what extent the respondent has experienced nine symptoms typical of depressive symptomatology over the past two weeks. The score options are “not at all” (scored 0), “some days” (1), “more than half the days” (2), or “almost every day” (3), resulting in a possible sum score ranging from 0 to 27. Higher composite score indicates more severe depression symptoms. The scale can also be scored categorically, with a cut-off score of 10 or higher being suggestive of clinical depression [67]. Cronbach’s alpha in the present study was 0.87 at baseline and 0.89 at follow-up for the continuous scale.

In the present study, both depression and anxiety were investigated as categorical outcome- and predictor variables to enhance this study’s clinical relevance.

### 4.2. Ethics

This study was approved by the Regional Committee for Medical and Health Research Ethics (REK number 249/110; approved 18 March 2019) and the Norwegian Center for Research Data (NSD number 758174; approved 1 April 2019). Participants had to be at least 16 years old and consent to take part in the study before being given access to the survey.

To increase the response rate, students participating in the follow-up study had the chance of winning one of ten gift cards of NOK 500 (corresponding to approximately USD 50) or one of five of Apple’s most recent iPhone models (iPhone Pro 12; Apple Inc., Cupertino, CA, USA).

### 4.3. Demographics

The survey also comprised demographic variables, including sex (“boy”/”girl”), date of birth, and parental education levels. Maternal and paternal educational levels were presented with the response option “primary/secondary school”, “high school”, “college/university < 4 years”, “college/university ≥ 4 years”, and “do not know”.

### 4.4. Statistical Analyses

All outcome analyses were performed using logistic regression analyses STATA (version 18.5, StataCorp., College Station, TX, USA). These analyses were performed using robust standard errors to adjust for clustering of observation within class levels. Additionally, potential changes in sleep patterns, the rate of students screening positive for anxiety and depression from baseline to follow-up, and changes in the use of prescribed sleep medications and caffeine intake, were investigated using paired samples t-tests (for continuous data) and McNemar’s tests (for categorical data) in IBM SPSS Statistics, version 29 (IBM Corp, Armonk, NY, USA).

In the longitudinal analyses, we investigated the associations between sleep-related parameters at baseline (hours of sleep duration on school and free days, chronic insomnia (no/yes), insomnia symptoms (sum score), social jetlag (<2 h versus ≥2 h), social jetlag (minutes), circadian preference (morning/intermediate/evening) and circadian preference (sum score)) and whether the students qualified for anxiety/depression (no/yes) at follow-up, while controlling for anxiety/depression (no/yes) at baseline.

Additionally, reverse analyses were performed to investigate whether anxiety and depression at baseline predicted changes in sleep duration (<8 h versus ≥8 h), chronic insomnia (no/yes), circadian preference (morning/intermediate/evening), and social jetlag (<2 h versus ≥2 h). In these analyses, morning and intermediate preferences were collapsed into one category and compared against evening-type preference.

Finally, the main analyses investigating whether the various sleep parameters at baseline predicted change in depression and anxiety from baseline to follow-up were repeated continuously to investigate whether the different sleep variables were also associated with change in depression and anxiety at symptom level. These analyses were performed using multiple linear regression analyses with robust standard errors on class levels. In these analyses, sleep duration was assessed both in minutes and hours, whereas social jetlag was assessed in minutes.

All analyses were repeated by including sex and maternal education as additional predictors. In the adjusted analyses, students unfamiliar with their mother’s educational level (*N* = 212) at baseline were excluded from the analyses.

### 4.5. Participant Flow

**Figure 1:**
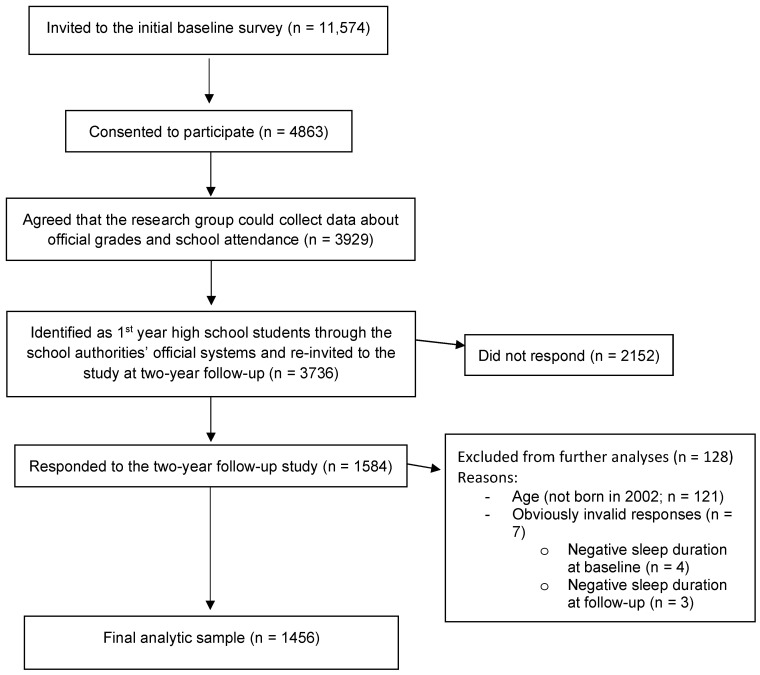
Flowchart of participants from initial invitation to final analytical sample.

According to the invited schools, the two counties had 11,574 registered 1st-year high school students. Of these, 4863 responded to the initial wave of the study (response rate: 42.0%). From these, 3736 students agreed that the researchers could link their responses to official registry-based data from the school authorities and to verify that they were registered as high school students, which were required to be invited to the follow-up survey. Among these, 1584 students (42.4% of the re-invited cohort) responded to the two-year follow-up survey. Of these, data from 121 students not born in 2002 were excluded from further analyses to avoid any age-confounding effects. Of the remaining 1463 participants, 4 were excluded from further analyses due to obviously invalid answers (i.e., negative sleep duration at baseline), whereas three additional participants were excluded due to negative sleep duration at follow-up. Thus, the final sample comprised 1456 participants (40.0% of the students invited to the follow-up survey). For complete flow of participants, please see Figure 1.

## 5. Conclusions

Longer sleep duration was associated with a reduced risk, whereas chronic insomnia and more severe insomnia symptoms were associated with increased risk of anxiety and depression at two-year follow-up among Norwegian adolescents. When circadian preference was investigated continuously, greater tendency toward morningness predicted a lower risk of depression and anxiety over time when controlled for sex and maternal education. Categorically, evening types had a higher risk of depression at follow-up than intermediate types, but the prospective risk of anxiety and depression did not otherwise differ across the circadian preference types. Social jetlag was unrelated to the prospective risk of anxiety and depression. In reverse analyses, anxiety and depression at baseline predicted a greater risk of chronic insomnia and lower risk of social jetlag ≥ 2 h over time, while baseline depression also predicted increased odds of evening-type preference from baseline to follow-up. The results indicate that targeting adolescent sleep problems may be beneficial for their mental health. The results also largely support the notion of bidirectional associations between sleep disturbances, anxiety and depression during adolescence.

## Figures and Tables

**Table 1:** Descriptive statistics of the longitudinal sample of 1456 Norwegian adolescents aged 16–17 years at baseline.

	Percentage	N
Sex		
Girl	61.4%	894
Boy	38.6%	562
Age		
16 years	64.3	936
17 years	35.7	520
Maternal educational level		
Junior high, high school or similar	3.8%	56
High school	18.7%	272
Higher education, <4 years	22.2%	323
Higher education, ≥4 years	40.7%	593
Don’t know	14.6%	212
Paternal educational level		
Junior high, high school or similar	5.3%	77
High school	25.1%	365
Higher education, <4 years	17.1%	249
Higher education, ≥4 years	34.1%	497
Don’t know	18.4%	268
Average units of caffeine per day		
None	43.8%	600
One	34.8%	476
Two	12.6%	172
Three or more	8.8%	121
Use of prescribed sleep medications		
Never	96.4%	1350
Less than weekly	1.1%	15
Weekly	0.8%	11
Daily	1.7%	24

Note. N = number of students.

**Table 2:** Baseline versus follow-up comparisons of sleep-related characteristics, anxiety, and depression in the final longitudinal sample.

	Descriptive Statistics		T Tests *			McNemar’s	*p* Value
	Baseline	Follow-Up	Mean Difference (SE Mean)	95% CI [Lower, Upper]	T	Test Statistics	
**Sleep patterns and insomnia**							
Sleep duration, school nights, hh:mm (*SD*)	6:49 (81 min)	6:55 (77 min)	−5.80 (2.29)	[−10.27, −1.29]	−2.52		0.012 ^a^
Sleep duration, free nights, hh:mm (*SD*)	8:37 (94 min)	8:35 (79 min)	0.663 (2.59)	[−4.42, 5.75]	0.26		0.798 ^a^
Social jetlag, hh:mm (*SD*)	2:36 (62 min)	2:28 (68 min)	7.73 (1.76)	[4.27, 11.19)	4.38		<0.001 ^a^
Social jetlag ≥ 2 hours (%)	73.9%	67.6%				16,687	<0.001 ^b^
BIS, total score (*M*, *SD*)	12.0 (7.9)	12.9 (8.3)	−0.92 (0.23)	[−1.37, −0.48]	−4.09		<0.001 ^a^
Chronic insomnia cases(%)	33.0%	35.4%				2267	<0.132 ^b^
**Depression**							
PHQ-9 (*M*, *SD*)	7.4 (5.5)	8.6 (6.0)	−1.24 (0.15)	[−1.52, −0.95]	−8.44		<0.001 ^a^
PHQ-9 above cut-off (%)	27.8%	35.9%				35,565	<0.001 ^b^
**Anxiety**							
GAD-7 (*M*, *SD*)	5.8 (4.7)	6.4 (5.2)	−0.66 (0.13)	[−0.91, −0.40)	−4.98		<0.001 ^a^
GAD-7, above cut-off (%)	27.6%	33.5%				19,563	<0.001 ^b^
**Circadian preference**							
MEQr, sum (*M*, *SD*)	12.9 (3.3)	13.2 (3.4)	−0.26 (0.07)	[−0.41, −0.12]	−3.54		<0.001 ^a^
MEQr, categorical (%)							
Morning type	9.7%	11.4%					
Intermediate type	53.0%	55.7%					
Evening type	37.3%	32.9%					

Note. BIS = Bergen Insomnia Scale; PHQ-9 = Patient Health Questionnaire-9; GAD-7 = Generalized Anxiety Disorder-7; rMEQ = the shortened version of the Morningness–Eveningness Questionnaire; *p* = *p* value of comparisons between baseline and follow-up. ^a^ = based on paired sample *t*-tests. ^b^ = based on McNemar’s test. * = based on minutes for sleep duration and social jetlag.

**Table 3:** Longitudinal associations between sleep duration, insomnia, circadian preference and social jetlag at baseline and the odds of anxiety and depression at follow-up among Norwegian high school students aged 16–17 years at baseline.

	Adjusted for Baseline Anxiety/Depression	Additional Adjustment for Sex and Maternal Education
	Odds Ratio (95% CI)	Odds Ratio (95% CI)
	**Anxiety**	**Anxiety**
Sleep duration (hours), school nights	**0.84 (0.77, 0.93)**	**0.87 (0.78, 0.96)**
Sleep duration (hours), free nights	0.93 (0.86, 1.01)	0.93 (0.85, 1.02)
Chronic insomnia		
No	1 (ref)	1 (ref)
Yes	**1.86 (1.43, 2.43)**	**1.92 (1.41, 2.62)**
Insomnia symptoms	**1.06 (1.04, 1.08)**	**1.07 (1.04, 1.09)**
Circadian preference, continuous	0.965 (0.928, 1.004)	**0.955 (0.914, 0.999)**
Circadian preference, categorical		
Morning	0.85 (0.57, 1.26)	0.77 (0.49, 1.21)
Intermediate	1 (ref)	1 (ref)
Evening	1.12 (0.87, 1.44)	1.13 (0.85, 1.51)
Social jetlag, minutes	1.001 (0.998, 1.003)	1.000 (0.998, 1.003)
Social jetlag ≥ 2 h		
<2 h	1 (ref)	1 (ref)
≥2 h	0.98 (0.74, 1.31)	0.95 (0.69, 1.30)
	**Depression**	**Depression**
Sleep duration (hours), school nights	**0.82 (0.75, 0.89)**	**0.81 (0.73, 0.89)**
Sleep duration (hours), free nights	0.99 (0.92, 1.07)	0.96 (0.88, 1.05)
Chronic insomnia		
No	1 (ref)	1 (ref)
Yes	**1.82 (1.36, 2.44)**	**2.02 (1.46, 2.78)**
Insomnia symptoms	**1.07 (1.05, 1.09)**	**1.07 (1.05, 1.09)**
Circadian preference, continuous	**0.949 (0.914, 0.986)**	**0.943 (0.905, 0.984)**
Circadian preference, categorical		
Morning	0.80 (0.52, 1.23)	0.93 (0.60, 1.44)
Intermediate	1 (ref)	1 (ref)
Evening	1.33 (0.998, 1.76)	**1.42 (1.05**, **1.94)**
Social jetlag, minutes	1.001 (0.999, 1.003)	1.001 (0.999, 1.003)
Social jetlag ≥ 2 h		
<2 h	1 (ref)	1 (ref)
≥2 h	1.04 (0.77, 1.40)	1.04 (0.75, 1.43)

Note. Significant sleep predictors in bold. Anxiety yes = total score ≥ 8 on the Generalized Anxiety Disorder-7; depression yes = total score ≥ 10 on the Patient Health Questionnaire-9; insomnia symptoms = continuous total score on the Bergen Insomnia Scale; chronic insomnia = insomnia symptoms above cut-off on the Bergen Insomnia Scale; rMEQ = The shortened version of the Morningness–Eveningness Questionnaire; CI = 95% confidence intervals; ref = reference category.

**Table 4:** Anxiety and depression as predictors of sleep duration ≥ 8 h, chronic insomnia, late circadian preference, and social jetlag ≥ 2 h at follow-up, controlled for the corresponding sleep parameters at baseline.

	Adjusted for Corresponding Sleep Parameter at Baseline	Additional Adjustment for Sex and Maternal Education
	Odds Ratio (95% CI)	Odds Ratio (95% CI)
	Sleep duration ≥ 8 h, school nights	Sleep duration ≥ 8 h, school nights
Anxiety		
No	1 (ref)	1 (ref)
Yes	0.94 (0.67, 1.32)	0.91 (0.62, 1.35)
Depression		
No	1 (ref)	1 (ref)
Yes	0.82 (0.57, 1.18)	0.81 (0.54, 1.23)
	Sleep duration ≥ 8 h, free nights	Sleep duration ≥ 8 h, free nights
Anxiety		
No	1 (ref)	1 (ref)
Yes	0.94 (0.71, 1.26)	0.93 (0.68, 1.28)
Depression		
No	1 (ref)	1 (ref)
Yes	0.93 (0.70, 1.25)	0.88 (0.64, 1.21)
	Chronic insomnia	Chronic insomnia
Anxiety		
No	1 (ref)	1 (ref)
Yes	**1.91 (1.46, 2.49)**	**1.84 (1.37, 2.48)**
Depression		
No	1 (ref)	1 (ref)
Yes	**2.00 (1.52, 2.64)**	**1.74 (1.27, 2.38)**
	Evening preference	Evening preference
Anxiety		
No	1 (ref)	1 (ref)
Yes	1.25 (0.93, 1.69)	1.30 (0.94, 1.79)
Depression		
No	1 (ref)	1 (ref)
Yes	**1.43 (1.05, 1.94)**	**1.40 (1.01, 1.96)**
	Social jetlag ≥ 2 h	Social jetlag ≥ 2 h
Anxiety		
No	1 (ref)	1 (ref)
Yes	**0.69 (0.53, 0.91)**	**0.74 (0.55, 0.997)**
Depression		
No	1 (ref)	1 (ref)
Yes	**0.69 (0.52, 0.92)**	**0.66 (0.48, 0.90)**

Note. Significant anxiety/depression predictors in bold. Anxiety yes = total score ≥ 8 on the Generalized Anxiety Disorder-7; depression yes = total score ≥ 10 on the Patient Health Questionnaire-9; chronic insomnia = insomnia symptoms above cut-off on the Bergen Insomnia Scale; evening preference = scores > 17 on the rMEQ; Odds ratio; CI = 95% confidence intervals (lower, upper); ref. = reference category.

## Data Availability

Data may be available upon request.

## Supplementary Materials

The following supporting information can be downloaded at: https://www.mdpi.com/article/10.3390/clockssleep7020026/s1.

## Abbreviations

The following abbreviations are used in this manuscript:

BIS	Bergen Insomnia Scale
GAD-7	Generalized Anxiety Disorder-7
PHQ-9	Patient Health Questionnaire-9
rMEQ	The reduced Morningness–Eveningness Questionnaire
WASO	Wake after sleep onset

## References

[1] Dahl R.E., Allen N.B., Wilbrecht L., Suleiman A.B. (2018). Importance of investing in adolescence from a developmental science perspective. Nature.

[2] Orben A., Tomova L., Blakemore S.J. (2020). The effects of social deprivation on adolescent development and mental health. Lancet Child Adolesc. Health.

[3] Paus T., Keshavan M., Giedd J.N. (2008). Why do many psychiatric disorders emerge during adolescence?. Nat. Rev. Neurosci..

[4] Paiva T., Gaspar T., Matos M.G. (2015). Sleep deprivation in adolescents: Correlations with health complaints and health-related quality of life. Sleep Med..

[5] Matos M.G., Gaspar T., Tomé G., Paiva T. (2016). Sleep variability and fatigue in adolescents: Associations with school-related features. Int. J. Psychol..

[6] Gradisar M., Gardner G., Dohnt H. (2011). Recent worldwide sleep patterns and problems during adolescence: A review and meta-analysis of age, region, and sleep. Sleep Med..

[7] Gariepy G., Danna S., Gobiņa I., Rasmussen M., Gaspar de Matos M., Tynjälä J., Janssen I.P., Kalman M.P., Villeruša A., Husarova D. (2020). How Are Adolescents Sleeping? Adolescent Sleep Patterns and Sociodemographic Differences in 24 European and North American Countries. J. Adolesc. Health.

[8] Jaworska N., MacQueen G. (2015). Adolescence as a unique developmental period. J. Psychiatry Neurosci..

[9] Kessler R.C., Berglund P., Demler O., Jin R., Merikangas K.R., Walters E.E. (2005). Lifetime prevalence and age-of-onset distributions of DSM-IV disorders in the National Comorbidity Survey Replication. Arch. Gen. Psychiatry.

[10] Hysing M., Sivertsen B., Nilsen S.A., Heradstveit O., Bøe T., Askeland K.G. (2023). Sleep and dropout from upper secondary school: A register-linked study. Sleep Health.

[11] Askeland K.G., Bøe T., Sivertsen B., Linton S.J., Heradstveit O., Nilsen S.A., Hysing M. (2022). Association of Depressive Symptoms in Late Adolescence and School Dropout. Sch. Ment. Health.

[12] Twenge J.M., Krizan Z., Hisler G. (2017). Decreases in self-reported sleep duration among U.S. adolescents 2009-2015 and association with new media screen time. Sleep Med..

[13] Crowley S.J., Wolfson A.R., Tarokh L., Carskadon M.A. (2018). An update on adolescent sleep: New evidence informing the perfect storm model. J. Adolesc..

[14] Carskadon M.A. (2011). Sleep in adolescents: The perfect storm. Pediatr. Clin. N. Am..

[15] Roenneberg T., Kuehnle T., Pramstaller P.P., Ricken J., Havel M., Guth A., Merrow M. (2004). A marker for the end of adolescence. Curr. Biol..

[16] Yip T., Wang Y., Xie M., Ip P.S., Fowle J., Buckhalt J. (2022). School Start Times, Sleep, and Youth Outcomes: A Meta-analysis. Pediatrics.

[17] Galan-Lopez P., Domínguez R., Gísladóttir T., Sánchez-Oliver A.J., Pihu M., Ries F., Klonizakis M. (2021). Sleep Quality and Duration in European Adolescents (The AdolesHealth Study): A Cross-Sectional, Quantitative Study. Children.

[18] Hirshkowitz M., Whiton K., Albert S.M., Alessi C., Bruni O., DonCarlos L., Hazen N., Herman J., Adams Hillard P.J., Katz E.S. (2015). National Sleep Foundation’s updated sleep duration recommendations: Final report. Sleep Health.

[19] Saxvig I.W., Bjorvatn B., Hysing M., Sivertsen B., Gradisar M., Pallesen S. (2021). Sleep in older adolescents. Results from a large cross-sectional, population-based study. J. Sleep Res..

[20] Wittmann M., Dinich J., Merrow M., Roenneberg T. (2006). Social jetlag: Misalignment of biological and social time. Chronobiol. Int..

[21] Mathew G.M., Li X., Hale L., Chang A.M. (2019). Sleep duration and social jetlag are independently associated with anxious symptoms in adolescents. Chronobiol. Int..

[22] Vitale J.A., Roveda E., Montaruli A., Galasso L., Weydahl A., Caumo A., Carandente F. (2015). Chronotype influences activity circadian rhythm and sleep: Differences in sleep quality between weekdays and weekend. Chronobiol. Int..

[23] Saxvig I.W., Evanger L.N., Pallesen S., Hysing M., Sivertsen B., Gradisar M., Bjorvatn B. (2021). Circadian typology and implications for adolescent sleep health. Results from a large, cross-sectional, school-based study. Sleep Med..

[24] Henderson S.E.M., Brady E.M., Robertson N. (2019). Associations between social jetlag and mental health in young people: A systematic review. Chronobiol. Int..

[25] Tamura N., Okamura K. (2024). Longitudinal course and outcome of social jetlag in adolescents: A 1-year follow-up study of the adolescent sleep health epidemiological cohorts. J. Sleep Res..

[26] Sateia M.J. (2014). International Classification of Sleep Disorders.

[27] de Zambotti M., Goldstone A., Colrain I.M., Baker F.C. (2018). Insomnia disorder in adolescence: Diagnosis, impact, and treatment. Sleep Med. Rev..

[28] Riemann D., Espie C.A., Altena E., Arnardottir E.S., Baglioni C., Bassetti C.L.A., Bastien C., Berzina N., Bjorvatn B., Dikeos D. (2023). The European Insomnia Guideline: An update on the diagnosis and treatment of insomnia 2023. J. Sleep Res..

[29] Bjorvatn B., Jernelöv S., Pallesen S. (2021). Insomnia—A Heterogenic Disorder Often Comorbid With Psychological and Somatic Disorders and Diseases: A Narrative Review With Focus on Diagnostic and Treatment Challenges. Front. Psychol..

[30] Mai E., Buysse D.J. (2008). Insomnia: Prevalence, Impact, Pathogenesis, Differential Diagnosis, and Evaluation. Sleep Med. Clin..

[31] Johnson E.O., Roth T., Schultz L., Breslau N. (2006). Epidemiology of DSM-IV insomnia in adolescence: Lifetime prevalence, chronicity, and an emergent gender difference. Pediatrics.

[32] Morin C.M., Bélanger L., LeBlanc M., Ivers H., Savard J., Espie C.A., Mérette C., Baillargeon L., Grégoire J.P. (2009). The natural history of insomnia: A population-based 3-year longitudinal study. Arch. Intern. Med..

[33] Yuksel D., Kiss O., Prouty D.E., Baker F.C., de Zambotti M. (2022). Clinical characterization of insomnia in adolescents—An integrated approach to psychopathology. Sleep Med..

[34] Harvey A.G. (2002). A cognitive model of insomnia. Behav. Res. Ther..

[35] Blank M., Zhang J., Lamers F., Taylor A.D., Hickie I.B., Merikangas K.R. (2015). Health correlates of insomnia symptoms and comorbid mental disorders in a nationally representative sample of US adolescents. Sleep.

[36] Baldini V., Gnazzo M., Rapelli G., Marchi M., Pingani L., Ferrari S., De Ronchi D., Varallo G., Starace F., Franceschini C. (2024). Association between sleep disturbances and suicidal behavior in adolescents: A systematic review and meta-analysis. Front. Psychiatry.

[37] Abdulghani H.M., Alrowais N.A., Bin-Saad N.S., Al-Subaie N.M., Haji A.M., Alhaqwi A.I. (2012). Sleep disorder among medical students: Relationship to their academic performance. Med. Teach..

[38] Harvey A.G. (2001). Insomnia: Symptom or diagnosis?. Clin. Psychol. Rev..

[39] Bacaro V., Miletic K., Crocetti E. (2024). A meta-analysis of longitudinal studies on the interplay between sleep, mental health, and positive well-being in adolescents. Int. J. Clin. Health Psychol..

[40] Alvaro P.K., Roberts R.M., Harris J.K. (2013). A Systematic Review Assessing Bidirectionality between Sleep Disturbances, Anxiety, and Depression. Sleep.

[41] Fang H., Tu S., Sheng J., Shao A. (2019). Depression in sleep disturbance: A review on a bidirectional relationship, mechanisms and treatment. J. Cell. Mol. Med..

[42] Narmandakh A., Roest A.M., Jonge P., Oldehinkel A.J. (2020). The bidirectional association between sleep problems and anxiety symptoms in adolescents: A TRAILS report. Sleep Med..

[43] Haraden D.A., Mullin B.C., Hankin B.L. (2017). The relationship between depression and chronotype: A longitudinal assessment during childhood and adolescence. Depress. Anxiety.

[44] Alvaro P.K., Roberts R.M., Harris J.K., Bruni O. (2017). The direction of the relationship between symptoms of insomnia and psychiatric disorders in adolescents. J. Affect. Disord..

[45] Li Y., Zhou Y., Ru T., Niu J., He M., Zhou G. (2021). How does the COVID-19 affect mental health and sleep among Chinese adolescents: A longitudinal follow-up study. Sleep Med..

[46] Magnusdottir S., Magnusdottir I., Gunnlaugsdottir A.K., Hilmisson H., Hrolfsdottir L., Paed A. (2024). Sleep duration and social jetlag in healthy adolescents. Association with anxiety, depression, and chronotype: A pilot study. Sleep Breath..

[47] Shanahan L., Copeland W.E., Angold A., Bondy C.L., Costello E.J. (2014). Sleep problems predict and are predicted by generalized anxiety/depression and oppositional defiant disorder. J. Am. Acad. Child Adolesc. Psychiatry.

[48] Gradisar M., Kahn M., Micic G., Short M., Reynolds C., Orchard F., Bauducco S., Bartel K., Richardson C. (2022). Sleep’s role in the development and resolution of adolescent depression. Nat. Rev. Psychol..

[49] Bacaro V., Carpentier L., Crocetti E. (2023). Sleep Well, Study Well: A Systematic Review of Longitudinal Studies on the Interplay between Sleep and School Experience in Adolescence. Int. J. Environ. Res. Public Health.

[50] Johnson E.O., Roth T., Breslau N. (2006). The association of insomnia with anxiety disorders and depression: Exploration of the direction of risk. J. Psychiatr. Res..

[51] Arnesen I.B., Bjorvatn B., Pallesen S., Waage S., Gradisar M., Wilhelmsen-Langeland A., Saxvig I.W. (2025). Insomnia in adolescent epidemiological studies: To what extent can the symptoms be explained by circadian factors?. Chronobiol. Int..

[52] Kallestad H., Hansen B., Langsrud K., Ruud T., Morken G., Stiles T.C., Gråwe R.W. (2011). Differences between patients’ and clinicians’ report of sleep disturbance: A field study in mental health care in Norway. BMC Psychiatry.

[53] Giannotti F., Cortesi F., Sebastiani T., Ottaviano S. (2002). Circadian preference, sleep and daytime behaviour in adolescence. J. Sleep Res..

[54] Randler C. (2011). Age and Gender Differences in Morningness–Eveningness During Adolescence. J. Genet. Psychol..

[55] Bowers J.M., Moyer A. (2017). Effects of school start time on students’ sleep duration, daytime sleepiness, and attendance: A meta-analysis. Sleep Health.

[56] Askeland K.G., Bøe T., Lundervold A.J., Stormark K.M., Hysing M. (2020). The Association Between Symptoms of Depression and School Absence in a Population-Based Study of Late Adolescents. Front. Psychol..

[57] Finning K., Ukoumunne O.C., Ford T., Danielson-Waters E., Shaw L., Romero De Jager I., Stentiford L., Moore D.A. (2019). Review: The association between anxiety and poor attendance at school—A systematic review. Child Adolesc. Ment. Health.

[58] Cortina J.M. (1993). What is coefficient alpha? An examination of theory and applications. J. Appl. Psychol..

[59] Danielsson K., Sakarya A., Jansson-Fröjmark M. (2019). The reduced Morningness-Eveningness Questionnaire: Psychometric properties and related factors in a young Swedish population. Chronobiol. Int..

[60] Roenneberg T., Wirz-Justice A., Merrow M. (2003). Life between clocks: Daily temporal patterns of human chronotypes. J. Biol. Rhythm..

[61] Adan A., Almirall H. (1991). Horne & Östberg morningness-eveningness questionnaire: A reduced scale. Personal. Individ. Differ..

[62] Horne J.A., Ostberg O. (1976). A self-assessment questionnaire to determine morningness-eveningness in human circadian rhythms. Int. J. Chronobiol..

[63] Pallesen S., Bjorvatn B., Nordhus I.H., Sivertsen B., Hjørnevik M., Morin C.M. (2008). A new scale for measuring insomnia: The Bergen Insomnia Scale. Percept. Mot. Ski..

[64] Bay T., Ergun A. (2018). Validity and reliability of Bergen Insomnia Scale (BIS) among adolescents. Clin. Exp. Health Sci..

[65] Spitzer R.L., Kroenke K., Williams J.B., Löwe B. (2006). A brief measure for assessing generalized anxiety disorder: The GAD-7. Arch. Intern. Med..

[66] Brattmyr M., Lindberg M.S., Solem S., Hjemdal O., Havnen A. (2022). Factor structure, measurement invariance, and concurrent validity of the Patient Health Questionnaire-9 and the Generalized Anxiety Disorder scale-7 in a Norwegian psychiatric outpatient sample. BMC Psychiatry.

[67] Kroenke K., Spitzer R.L., Williams J.B. (2001). The PHQ-9: Validity of a brief depression severity measure. J. Gen. Intern. Med..

[68] Spitzer R.L., Kroenke K., Williams J.B. (1999). Validation and utility of a self-report version of PRIME-MD: The PHQ primary care study. Primary Care Evaluation of Mental Disorders. Patient Health Questionnaire. JAMA.

[69] Burdzovic Andreas J., Brunborg G.S. (2017). Depressive Symptomatology among Norwegian Adolescent Boys and Girls: The Patient Health Questionnaire-9 (PHQ-9) Psychometric Properties and Correlates. Front. Psychol..

[70] Wisting L., Johnson S.U., Bulik C.M., Andreassen O.A., Rø Ø., Bang L. (2021). Psychometric properties of the Norwegian version of the Patient Health Questionnaire-9 (PHQ-9) in a large female sample of adults with and without eating disorders. BMC Psychiatry.

