# Supplementary material for: Associations Between Sleep Patterns, Circadian Preference, and Anxiety and Depression: A Two-Year Prospective Study Among Norwegian Adolescents
Source: Clocks Sleep. 2025 May 27;7(2):26. doi: 10.3390/clockssleep7020026 (PMC12191514; doi:10.3390/clockssleep7020026)
Supplement: Supplementary file 1 [file clockssleep-07-00026-s001.zip › clockssleep-3413501-supplementary.pdf]

Supplementary Materials

Table S1. Baseline associations between demographics and sleep characteristics and the odds of anxiety and depression among 1,456 Norwegian high school students aged 16-17 years.

	**Unadjusted**	**Adjusted for sex and maternal education**
	**Odds ratio (95% CI)**	**Odds ratio (95% CI)**
	**Anxiety**	**Anxiety**
Sex		
Girl	1 (ref)	
Boy	**0.34 (0.26, 0.45)**	
Maternal education		
Junior high or lower	.80 (0.39, 1.62)	
High school	1.18 (0.85, 1.64)	
College/university < 4 years	1.02 (0.74, 1.42)	
College/university ≥ 4 years	1 (ref)	
Sleep duration (hours), school nights	**0.71 (0.64, 0.77)**	**0.73 (0.66, 0.81)**
Sleep duration (hours), free nights	**0.87 (0.81, 0.94)**	**0.88 (0.81, 0.96)**
Chronic insomnia		
No	1 (ref)	1 (ref)
Yes	**4.02 (3.12, 5.18)**	**3.65 (2.79, 4.79)**
Insomnia symptoms	**1.13 (1.10, 1.15)**	**1.12 (1.09, 1.14)**
Circadian preference, continuous	**0.961 (0.924, 0.999)**	0.959 (0.918, 1.001)
Circadian preference, categorical		
Morning	1.10 (0.73, 1.68)	1.27 (0.81, 1.99)
Intermediate	1 (ref)	1 (ref)
Evening	1.30 (0.99, 1.70)	1.34 (0.99, 1.81)
Social jetlag, minutes	0.999 (0.997, 1.001)	1.001 (0.998, 1.003)
Social jetlag ≥ 2 hours		
< 2 hours	1 (ref)	1 (ref)
≥ 2 hours	0.84 (0.65, 1.09)	0.92 (0.69, 1.22)
	**Depression**	**Depression**
Sex		
Girl	1 (ref)	
Boy	**0.38 (0.29, 0.50)**	
Maternal education		
Junior high or lower	0.87 (0.42, 1.80)	
High school	1.32 (0.95, 1.83)	
College/university < 4 years	1.20 (0.89, 1.63)	
College/university ≥ 4 years (ref)	1 (ref)	
Sleep duration (hours), school nights	**0.67 (0.60, 0.74)**	**0.68 (0.60, 0.77)**
Sleep duration (hours), free nights	**0.83 (0.77, 0.90)**	**0.82 (0.75, 0.90)**
Chronic insomnia		

No	1 (ref)	1 (ref)
Yes	**6.25 (4.82, 8.09)**	**5.44 (4.11, 7.20)**
Insomnia symptoms	**1.17 (1.15, 1.19)**	**1.16 (1.14, 1.19)**
Circadian preference, continuous	**0.889 (0.852, 0.927)**	**0.885 (0.845, 0.926)**
Circadian preference, categorical		
Morning	**0.65 (0.42, 0.996)**	0.65 (0.41, 1.01)
Intermediate	1 (ref)	1 (ref)
Evening	**1.75 (1.34, 2.28)**	**1.78 (1.33, 2.38)**
Social jetlag, minutes	1.001 (0.999, 1.003)	1.002 (1.000, 1.004)
Social jetlag ≥ 2 hours		
< 2 hours	1 (ref)	1 (ref)
≥ 2 hours	0.92 (0.70, 1.20)	0.95 (0.71, 1.29)

Note. Significant sleep predictors are outlined in bold. Anxiety yes = total score ≥ 8 on the Generalized Anxiety Disorder-7; depression yes = total score ≥ 10 on the Patient Health Questionnaire-9; insomnia symptoms = total score on the Bergen Insomnia Scale; chronic insomnia = insomnia above cut-off on the Bergen Insomnia Scale; rMEQ = The shortened version of the Morningness-Eveningness Questionnaire; CI = 95% confidence intervals; ref. = reference category
